# Resting state functional connectivity patterns as biomarkers of treatment response to escitalopram in patients with major depressive disorder

**DOI:** 10.1007/s00213-021-05915-7

**Published:** 2021-09-03

**Authors:** Marieke A. G. Martens, Nicola Filippini, Catherine J. Harmer, Beata R. Godlewska

**Affiliations:** 1 Department of Psychiatry, University of Oxford, Oxford, UK; 2 Wellcome Center for Integrative Neuroimaging, University of Oxford, OX3 9DU Oxford, UK; 3 Oxford Centre for Human Brain Activity, Wellcome Centre for Integrative Neuroimaging, Department of Psychiatry, University of Oxford, Oxford, UK; 4 Oxford Health NHS Foundation Trust, Oxford, UK

**Keywords:** Major depressive disorder, Treatment biomarkers, Treatment response, Escitalopram, Resting-state fMRI, Resting-state networks, Independent component analysis

## Abstract

**Rational:**

With no available response biomarkers, matching an appropriate antidepressant to an individual can be a lengthy process. Improving understanding of processes underlying treatment responsivity in depression is crucial for facilitating work on response biomarkers.

**Objectives:**

To identify differences in patterns of pre-treatment resting-state functional connectivity (rsFC) that may underlie response to antidepressant treatment.

**Methods:**

After a baseline MRI scan, thirty-four drug-free patients with depression were treated with an SSRI escitalopram 10 mg daily for 6 weeks; response was defined as ≥ 50% decrease in Hamilton Depression Rating Scale (HAMD) score. Thirty-one healthy controls had a baseline clinical assessment and scan. Healthy participants did not receive treatment.

**Results:**

Twenty-one (62%) of patients responded to escitalopram. Treatment responsivity was associated with enhanced rsFC of the right fronto-parietal network (FPN)—with the posterior DMN, somatomotor network (SMN) and somatosensory association cortex. The lack of treatment response was characterized by reduced rsFC: of the bilateral FPN with the contralateral SMN, of the right FPN with the posterior DMN, and of the extended sensorimotor auditory area with the inferior parietal lobule (IPL) and posterior DMN. Reduced rsFC of the posterior DMN with IPL was seen in treatment responders, although only when compared with HC.

**Conclusions:**

The study supports the role of resting-state networks in response to antidepressant treatment, and in particular the central role of the frontoparietal and default mode networks.

**Supplementary Information:**

The online version contains supplementary material available at 10.1007/s00213-021-05915-7.

## Introduction

Identification of biomarkers allowing prediction of antidepressant response to individual drugs and allocation of the right treatment to a given patient is a key priority in psychiatric research. Currently, finding such treatment follows a ‘trial and error’ process, based on guidelines derived from the knowledge of populational efficacy of drugs; this process however does not necessarily work for a particular individual (Cipriani et al. 2018; Shinohara et al. 2019). In fact, only one-third of depressed patients respond to their first treatment, and finding an effective one often takes months or longer (Warden et al. 2007; Rush et al. 2009). Despite significant research efforts, clinically applicable biomarkers have not yet been identified.

Such research has been largely facilitated by the development of non-invasive in vivo imaging techniques, which put forward some potential biomarkers, such as increased activity of the pgACC as a general predictor of good treatment response (Fu et al. 2013; Godlewska et al. 2018). However, after years of research, it has become increasingly unlikely that an aberrant function of one particular structure in the brain could reliably predict treatment response. This is in line with depression being conceptualized as a network disorder, with meaningful aberrations related to the networks of brain structures rather than individual regions (Li et al. 2018).

Neuroimaging has been the key in the understanding of network activity. A commonly used method is an assessment of temporal correlation of the activity of brain regions—so-called functional connectivity (FC)—with functional magnetic resonance (fMRI) technique, either during task performance or when an individual is awake yet not engaged in any external tasks (so-called resting state). Assessment of resting-state functional connectivity (rsFC) has some important benefits: it is task independent; hence, results are not dependent on the selection of an appropriate task, data collection is relatively easy as it does not depend on the ability to perform a task, which may be impaired in depression, and it allows an assessment of networks predominantly activated during rest. Resting state analysis can be performed with different methods, all having one assumption in common: the definition of functional connectivity is based on a statistical dependency between time series, expressing co-activation of brain structures. Conventional methods of analyzing resting-state FC (rsFC) can roughly be divided into region-of-interest (ROI)–based and data-driven methods (Yang et al., 2020). While both approaches are beneficial, ROI-based methods require a pre-existing hypothesis as to the role of a region, and a choice of a ‘seed’ can be quite subjective with slightly different seeds giving different results. A seed-based analysis is furthermore restricted by having to predefine the region a priori which does not allow for explorative analysis. An alternative is defining nodes based on the data in a data-driven parcellation by, for example, independent component analysis (ICA) (Yang et al., 2020). A benefit of this method is that new regions can be discovered from which new hypotheses can be formulated.

Both types of analysis provided support for models of depression focused on dysfunctional communication between brain regions and helped to explore mechanisms of antidepressant action at the network level. Two prominent models focused on the role of inadequate cognitive control by the central executive network (CEN) over the affective and default mode networks (AN and DMN, respectively), resulting in inadequate top-down regulation of attention and emotion regulation in depressed patients regardless of their treatment status (Drevets 2001, Mayberg 2003, Wang et al. 2012, Kaiser et al., 2015, Zhong et al. 2016).

In line with these models, antidepressants, including a number of serotonin reuptake inhibitor (SSRIs), serotonin-norepinephrine reuptake inhibitor (SNRIs) and an norepinephrine reuptake inhibitor (NRI) reboxetine were shown, through both data- and hypothesis-driven analyses, to modulate disrupted network connectivity in the fronto-limbic networks and between the frontal and DMN regions (McCabe et al. 2011; Dichter et al. 2015; Vai et al. 2016; Brakowski et al. 2017), with potentially key role of the posterior DMN (Li et al. 2013; Posner et al. 2013). Some findings are promising as potential biomarkers, such as, for example, low connectivity between the lateral PFC and sgACC being a predictor of good treatment response, which was shown for both typical antidepressant drugs, such as an SSRI sertraline (Chin Fatt et al. 2020) and a new glutamatergic treatment ketamine (Gartner et al. 2019). SgACC connectivity may also play a role in non-pharmacological methods, such as TMS (Philip et al. 2018). At this point, however, there are no clinically useful biomarkers of response, and although the work on the proposed markers needs to continue, it is similarly important to explore brain connectivity in a data-driven manner, so that potentially relevant connections are not missed because of restriction related with an a priori selection of brain regions included in the analysis.

Therefore, this study employed a whole-brain data-driven independent component analysis, in order to explore differences in patterns of baseline (pre-treatment) resting-state functional connectivity (rsFC) in patients with major depressive disorder (MDD) who responded to 6 weeks of treatment with an SSRI escitalopram, patients who did not respond to such treatment and healthy volunteers.

## Methods

### Participants and study design

Patients with MDD were recruited through referral from clinicians and advertising, healthy controls through advertising. Thirty-nine patients with MDD and 32 healthy subjects gave written informed consent to take part in the study. Thirty-four patients (19F:15 M) completed both baseline fMRI scan and 6-week period of escitalopram treatment; 31 healthy subjects completed the scan (see Table 1 for demographic information). In the remaining 5 patients and 1 healthy subject, relevant data were not available at the end of the treatment period (four patients dropped out before the 6-week assessment, scanning data from one patient were unavailable due to excessive motion and one healthy volunteer was not scanned due to safety concerns revealed at the scanning appointment). All participants were assessed with the Structured Clinical Interview for DSM-IV (Spitzer et al 1995) for the presence of current and past psychiatric disorders. The depressed patients met DSM-IV criteria for a primary diagnosis of major depressive disorder; exclusion criteria for both groups were as follows: past/current DSM-IV diagnosis of axis I disorder (other than MDD in patients), substance dependence as defined by DSM-IV, a clinically significant risk of suicidal behaviour, major somatic or neurological disorders, pregnancy or breast-feeding, any contra-indications to MR imaging, or concurrent medication that could alter emotional processing. Patients with contraindications to escitalopram treatment or treatment with psychotropic medication less than 3 weeks before the study (5 weeks for fluoxetine) were also excluded from the study. At baseline, 22 patients were drug-naïve and 12 patients were drug-free (for all patients: range 2–117 months, mean 32.2 months, SD 38.4 months). Previous treatments included sertraline, fluoxetine, escitalopram, paroxetine, venlafaxine, mirtazapine, trazodone, imipramine and lofepramine. All participants were right-handed. The study was approved by the Oxford Research Ethics Committee and performed in accordance with Declaration of Helsinki.

**Table 1:** Demographics and clinical scores presented as a mean ± standard deviation (SD) (except for gender). *R*, responders; *NR*, non-responders; *HC*, healthy controls; *HAMD*, Hamilton Depression Rating Scale; *BDI*, Beck Depression Inventory (BDI) (Beck et al. 1961); *STAI-T*, Spielberger’s State-Trait Anxiety Inventory–Trait

	Responders (R) *N* = 21	Non-responders (NR) *N* = 13	Statistics for R vs NR (independent samples *t*-test, exc. chi-square for gender*)		Healthy controls (HC) *N* = 31		Statistics for R vs NR vs HC (one-way ANOVA, exc. chi-square for gender*)		
			*p*	*t*	df		*p*	*F*	df
Age (years)	30.4 ± 11.7	30.1 ± 10.1	0.939	-0.077	32	30.3 ± 10.0	0.997	0.003	64
Gender	11F and 10 M	8F and 5 M	0.601	0.273*	1	18F and 13 M	0.858	0.306*	2
Age at onset (years)	25.1 ± 8.3	25.2 ± 11.3	0.968	0.040	32	NA	NA	NA	NA
Duration of current episode (months)	5.9 ± 6.1	8.8 ± 8.7	0.276	1.108	32	NA	NA	NA	NA
HAMD baseline	23.3 ± 4.9	22.9 ± 4.0	0.823	-0.226	32	0.4 ± 0.8	< 0.0001	381.5	64
HAMD at 6 weeks	4.5 ± 3.8	17.4 ± 5.1	< 0.0001	8.442	32	NA	NA	NA	NA
BDI baseline	31.6 ± 6.8	32.4 ± 5.5	0.735	0.341	32	0.8 ± 1.5	< 0.0001	358.0	64
BDI at 6 weeks	9.2 ± 7.3	24.2 ± 10.6	< 0.0001	4.899	32	NA	NA	NA	NA
STAI-Trait anxiety at baseline	60.0 ± 8.4	63.3 ± 10.3	0.321	1.009	32	29.2 ± 7.7	< 0.0001	116.1	64
STAI-Trait anxiety at 6 weeks	46.1 ± 14.1	56.9 ± 11.3	0.026	2.335	32	NA	NA	NA	NA

After the baseline fMRI scan, patients received 10 mg escitalopram each morning for 6 weeks without dose adjustment. Depressive severity and treatment response were measured using the 17-item Hamilton Depression Rating Scale (HAMD) (Hamilton 1960) and Beck Depression Inventory (BDI) (Beck et al. 1961), and anxiety levels were measured with Spielberger’s State-Trait Anxiety Inventory–Trait (STAI-T) (Spielberger 1989) at baseline and after 6 weeks of treatment. The current analysis focuses on how baseline differences in rsFC were able to predict clinical response at week 6 of treatment. After the study was completed, all patients were offered treatment openly with escitalopram following usual clinical practice. Clinical response to the SSRI was defined as a reduction in HAMD of 50% or more from baseline after 6 weeks of treatment (Angst et al. 1993). Healthy control subjects followed the same protocol; however, they did not receive any medication. Resting state data acquisition was performed with participants having their eyes closed.

### Data acquisition

fMRI data were acquired on a 3 T Siemens Magnetom TIM TRIO scanner (Siemens AG) at the University of Oxford Centre for Clinical Magnetic Resonance Research (OCMR). A total of 180 volumes of resting-state fMRI (rs-fMRI) were acquired with a voxel resolution of 3 × 3 × 3.5 mm, repetition time (TR)/echo time (TE)/flip angle (FA) = 2000 ms/28 ms/89°, duration time: 6 min 4 s. T1-weighted structural images were acquired using a magnetization prepared rapid acquisition by gradient echo sequence (MPRAGE) with a voxel resolution 1.0 × 1.0 × 1.0 mm on a 208 × 256 × 200 grid, TR/TE/inversion time (TI) = 2040 ms/4.68 ms/900 ms. Gradient echo phase and magnitude field maps to correct for distortion were also acquired with voxel resolution of 3.5 × 3.5 × 3.0 mm, TR/TE1/TE2/FA = 488 ms/5.19 ms/7.65 ms/60°.

### Resting-state fMRI preprocessing and statistical analysis

Rs-fMRI data was analyzed using FMRIB software library (FSL version 6.6.1, FMRIB Analysis group, Oxford University, UK) (Jenkinson et al., 2012). Questionnaire data was analyzed with Statistical Package for the Social Sciences (SPSS 25) (IBM Corporation, Armonk, NY USA).

Briefly, single-subject pre-processing consisted of motion correction, brain extraction of structural and functional data, unwarping of EPI data using the acquired field maps, co-registration of EPI data to the structural scan using boundary-based registration, and normalization into MNI standard space using both linear and nonlinear image registration tools. Temporal filtering with a high-pass filter cut-off of 150 s (0.007 Hz) and spatial smoothing with a Gaussian kernel of full-width at half maximum of 6 mm were applied.

Individual subject independent component analysis (ICA) was carried out using Multivariate Exploratory Linear Optimized Decomposition into Independent Components (MELODIC) (Beckmann et al. 2005). Single-session, independent components (ICs) were automatically classified into signal or noise using FMRIB’s ICA-based Xnoiseifier (FIX), with a standard training dataset (Griffanti et al. 2014; Salimi-Khorshidi et al. 2014). De-noised functional data were temporally concatenated across subjects and decomposed into ICs using MELODIC. Dimensionality estimation for group maps was set to 25 IC maps, which is an approximate average of the individual IC maps thresholds as determined by spatial mixture modelling. They were identified as either belonging to the most frequently reported major RSNs (Smith et al., 2009), or reflecting movement, physiological or scanner noise This was done by visual inspection, independently by MM and BG, and subsequently consulted with NF, and additionally through a comparison to previously published maps (Smith et al., 2009) using Pearson spatial cross-correlation. Next, the set of spatial maps from the group-average analysis was used to generate subject-specific versions of the spatial maps, and associated timeseries, using dual regression (Beckmann et al. 2009; Filippini et al. 2009). First, for each subject, the group-average set of spatial maps was regressed (as spatial regressors in a multiple regression) into the subject’s 4D space–time dataset. This resulted in a set of subject-specific timeseries, one per group-level spatial map. Next, those timeseries were regressed (as temporal regressors, again in a multiple regression) into the same 4D dataset, resulting in a set of subject-specific spatial maps, one per group-level spatial map. We then tested for statistically significant differences between the groups using FSL’s randomize permutation-testing tool (5000 permutations). Cluster-based thresholding was applied using Threshold-Free-Cluster-Enhancement (TFCE) approach and a family-wise-error corrected cluster significance threshold of *p* < 0.05 applied to the suprathreshold clusters (Nichols and Holmes 2002, Smith and Nichols, 2009).

Structural images were used as additional covariates on a voxel-by-voxel basis to interrogate rs-fMRI data. GM images of each subject were extracted using FMRIB’s Automated Segmentation Tool (http://fsl.fmrib.ox.ac.uk/fsl/fslwiki/FAST/), registered to standard space, smoothed to match the intrinsic smoothness of the rs-fMRI data, voxel-wise demeaned across all subjects in both groups together and added as a confounding regressor (nuisance) to the GLM design matrix used to analyze rs-fMRI data. This was done using an FSL script ‘feat_GM_prepare’, which estimates the smoothness of the functional data from the melodic folder and matches the fMRI data. Adding GM maps reduces variance in the data due to potentially confounding anatomical differences between subjects (Oakes et al. 2007).

Additionally, using Pearson’s r correlation, we performed correlational analysis between identified functional connectivity measures (parameter estimates) and clinical measures: BDI score at baseline and its percent change over the time of treatment (total score and score on BDI subscales: affective, cognitive and somatic) (Buckley et al. 2001), STAI-Trait total score and length of the current episode. Details of the items allocated to individual BDI subscales are presented in Supplementary Table 1.

## Results

### Clinical and demographic data

Twenty-one out of 34 (62%) patients responded to 6 weeks of treatment with escitalopram. Responders and non-responders did not differ with respect to gender, age, baseline depression severity, baseline trait anxiety, age at depression onset and duration of current episode (see Table 1 for details). Healthy controls, as expected, differed from patients in terms of depression and anxiety scores; there were no significant differences in terms of gender and age (Table 1).

### Resting-state fMRI imaging analysis

**Fig. 1:**
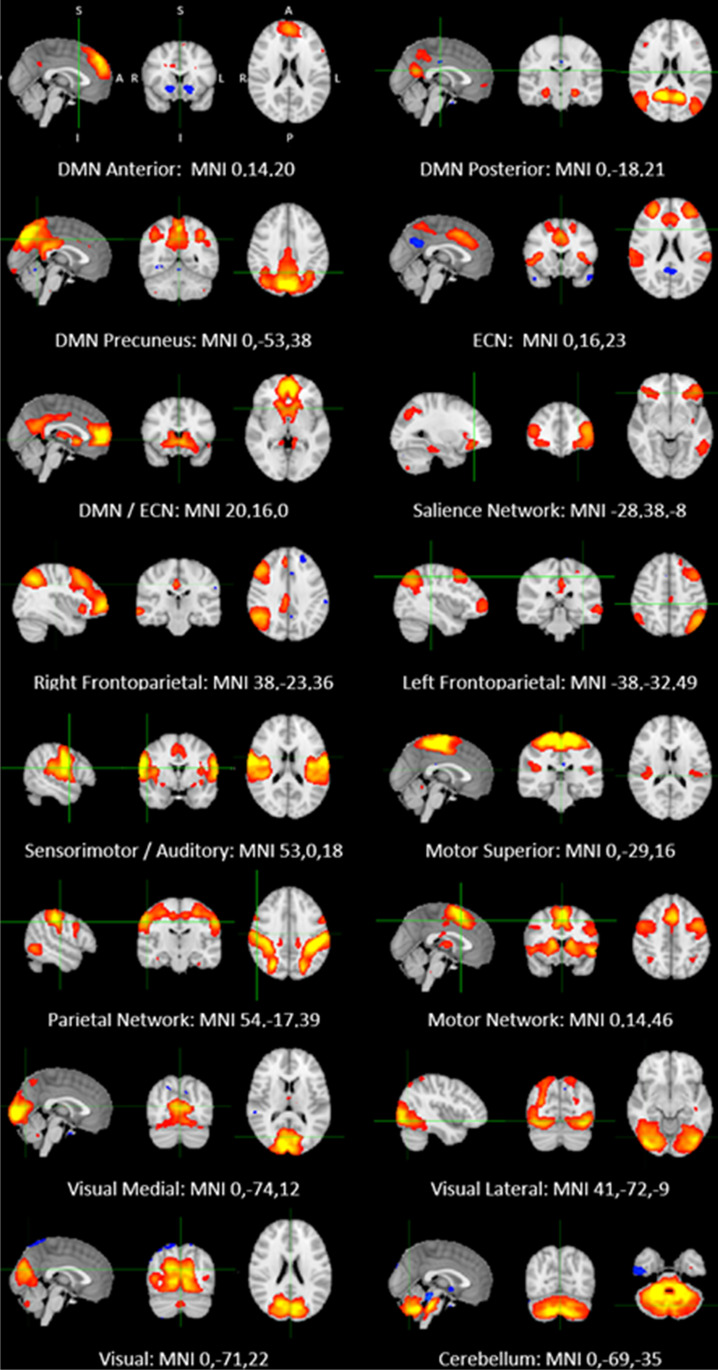
Resting-state networks identified in the study. Axial, coronal and sagittal slices for the main resting-state networks detected, overlaid onto the standard MNI brain. All maps thresholded at *Z* = 3

All individual datasets were quality checked. No participant exceeded the cut-off for excursion from the initial head position, 1.5 mm. Out of 25 ICs extracted from the temporal concatenation, 16 components were identified as resting-state networks (RSNs) of interest corresponding to previously identified canonical RNSs (Yeo et al. 2011). The other components represented noise, which was not excluded by the artefact rejection algorithm (FIX) and included artefacts resulting from physiological noise (arteries, veins and CSF), head motion and scanner artefacts. All RSNs of interest are displayed in Fig. 1.

### Independent component analysis

Nonparametric permutation analysis revealed significant group differences in baseline functional connectivity between (1) treatment responders (R) and non-responders (NR), (2) NR and healthy controls (HC), and (3) R and HC. Differences in resting-state networks were also observed when comparing MDD patients as a single group, regardless of their response status (R or NR), and HC; however, these were driven by either R or NR and therefore we do not describe them in detail.

**Fig. 2:**
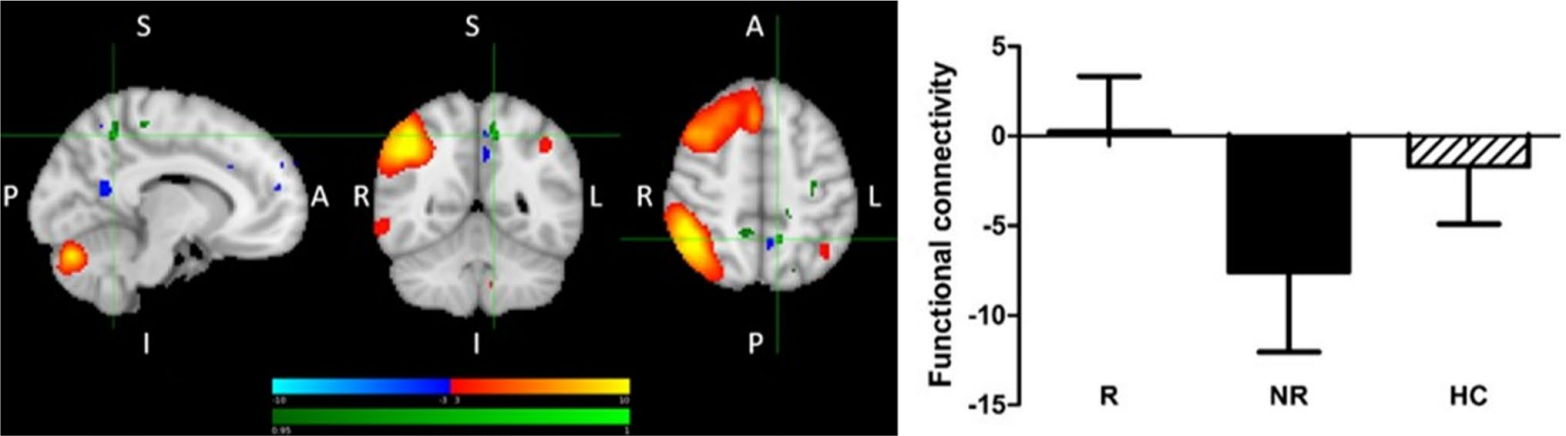
Greater pre-treatment functional connectivity in treatment responders compared with non-responders between the right fronto-parietal network and bilateral precuneus, left precentral and postcentral gyri and somatosensory association cortex. Red-yellow: regions with correlated BOLD signal time-course; blue: regions with anti-correlated BOLD signal time-course; green: group differences between responders and non-responders. The graph shows parameter estimates (beta-values) extracted from the cluster of significant difference between the groups. R > NR, right frontoparietal network, mean left and right precuneus. MNI coordinates of cursor =  − 10, − 52, 50. Results are shown TFCE-corrected with a family-wise error cluster significance level of *p* < 0.05. Error bars denote standard deviation

**Fig. 3:**
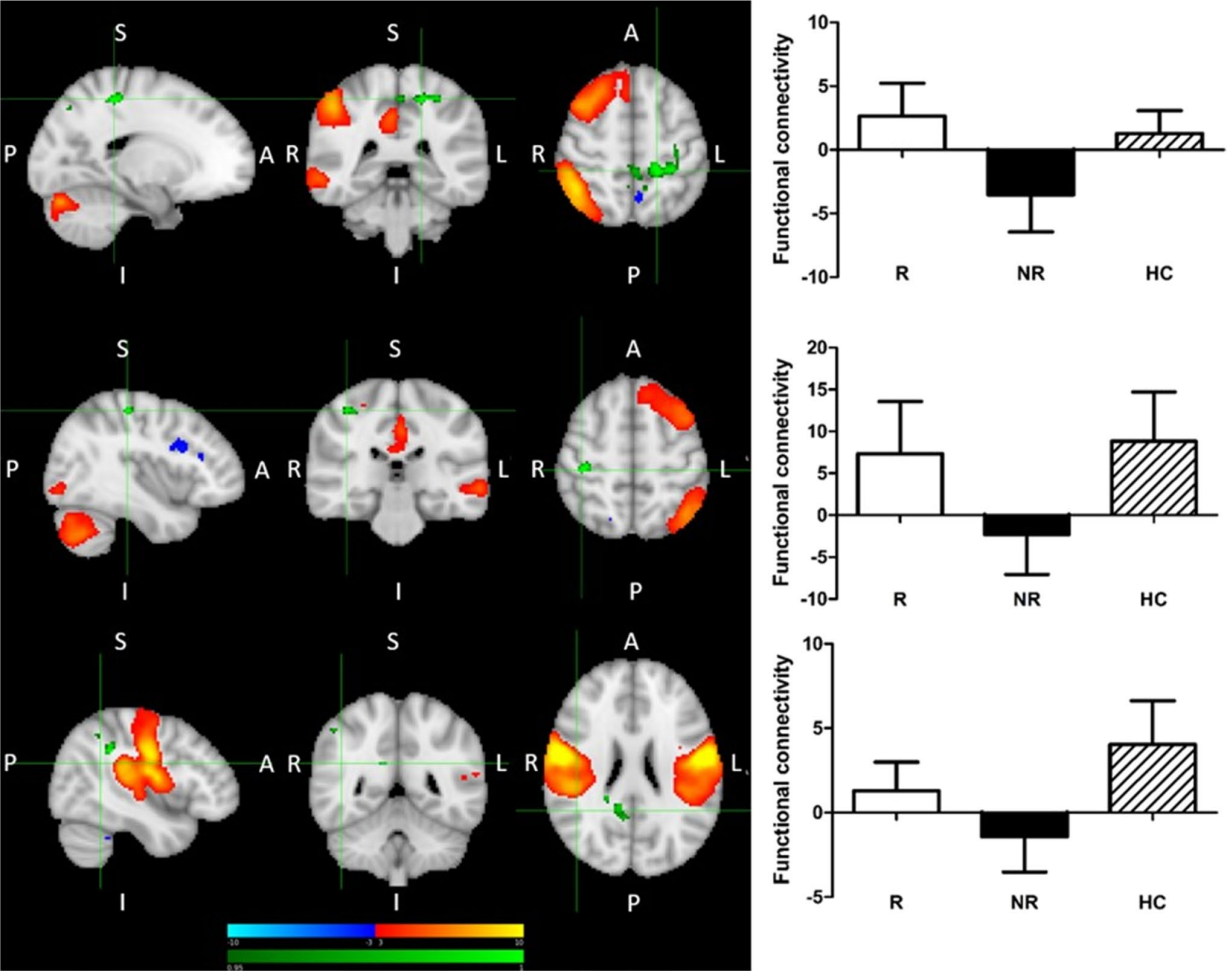
Increased pre-treatment functional connectivity between: (a) right fronto-parietal network and left postcentral/precentral gyrus; R > NR & HC > NR; MNI coordinates of cursor =  − 18, − 38, 54; (b) left fronto-parietal network and right postcentral/precentral gyrus; HC > NR; MNI coordinates of cursor = 40, − 28, 54; (c) sensorimotor-auditory network and angular gyrus/supramarginal gyrus/precuneous cortex /PCC; HC > NR; MNI coordinates of cursor = 43, − 49, 24. Red-yellow: regions with correlated BOLD signal time-course; blue: regions with anti-correlated BOLD signal time-course; green: group differences. The graph shows parameter estimates extracted (beta-values) from the cluster of significant difference between the groups. Results are shown TFCE-corrected with a family-wise error cluster significance level of *p* < 0.05. Error bars denote standard deviation

**Fig. 4:**
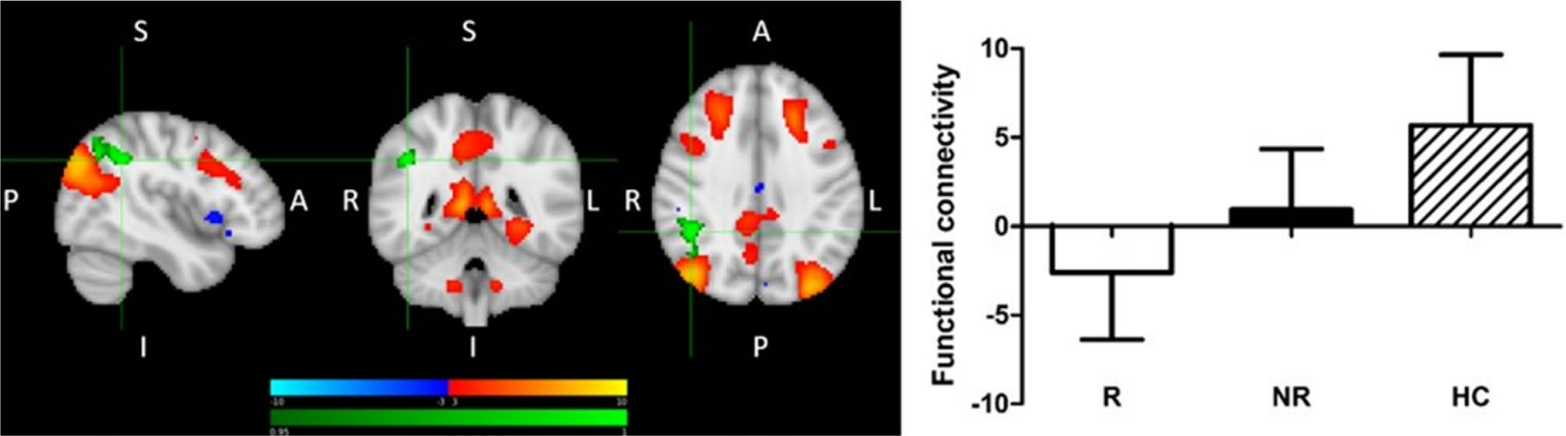
Decreased pre-treatment functional connectivity in responders compared with healthy volunteers between the default mode network and right angular gyrus/supramarginal gyrus. Red-yellow: regions with correlated BOLD signal time-course; blue: regions with anti-correlated BOLD signal time-course; green: group differences. The graph shows parameter estimates (beta-values) extracted from the cluster of significant difference between the groups. MNI coordinates of cursor = 44, − 46, 36. Results are shown TFCE-corrected with a family-wise error cluster significance level of *p* < 0.05. Error bars denote standard deviation

(1) Treatment R, as compared to NR, showed greater pre-treatment FC between the right fronto-parietal network (FPN) and bilateral precuneus, as well as between the right FPN and left precentral and postcentral gyri and somatosensory association cortex (Fig. 2). (2) Treatment NR, as compared to HC, showed reduced baseline FC of the right and left FPN with contralateral precentral and postcentral gyri, and reduced baseline FC of a somatomotor-auditory component including the sensorimotor network (SMN) and other sensory and motor areas (auditory cortex, posterior insula, central and parietal operculum, midcingulate cortex—MCC, and supplementary motor area—SMA) with right precuneus/posterior cingulate cortex (PCC), as well as between this sensorimotor-auditory component and right angular and supramarginal gyri (ANG and SMG, respectively) (Fig. 3a-c). Statistically significant differences were observed between all patients with MDD and HC for all of the above connections except the right FPN. The results were driven by the NR group, and no statistically significant differences were noted between R and HC, or R and NR. (3) Treatment R, as compared to HC, was characterized by reduced baseline FC between the default mode network (DMN) and right ANG/SMG (Fig. 4). Statistically significant differences were observed between all patients with MDD and HC. The results were driven by the R group, with no statistically significant differences between NR and HC, or R and NR. Details of significant functional connections between resting-state networks and individual brain regions are presented in Table 2, while statistical parametric maps for the above contrasts, as well as parameter estimates extracted from the clusters of significant difference between the groups, are displayed in Figs. 2, 3 and 4.

**Table 2:** Significant functional connections (temporal correlations) between resting-state networks and individual brain regions. Individual clusters identified within each contrast shown. *R*, responders; *NR*, non-responders; *HC*, healthy controls

Contrast	Network	Cluster	Cluster size (number of voxels)	Peak voxel	Pmax value
R > NR	Right fronto-parietal network (FPN)	Left postcentral gyrus	259	− 18, − 38, 54	0.978
		Left precentral gyrus (extending into left precuneus)	48	− 2, − 38, 54	0.965
		Left precuneus	29	− 10, − 52, 50	0.961
		Right precuneus	27	10, − 48, 50	0.957
		Left somatosensory association cortex	28	− 20, − 74, 46	0.964
R > NR & HC > NR	Right fronto-parietal network (FPN)	Left postcentral gyrus	65	− 12, − 34, 58	0.973
		Left precentral and postcentral gyri	44	− 30, − 26, 44	0.972
HC > NR	Left fronto-parietal network (FPN)	Right postcentral and precentral gyri	44	40, − 28, 54	0.985
HC > NR	Sensorimotor-auditory network	Right supramarginal and angular gyri	46	44, − 42, 34	0.970
		Right angular gyrus	45	48, − 52, 46	0.969
		Right precuneus and posterior cingulate cortex	33	12, − 46, 24	0.964
HC > R	Default mode network (DMN)	Right angular and supramargial gyri	309	44, − 46, 36	0.994

Although the two groups, responders and non-responders, did not differ in terms of baseline HAMD score (Table 1), in order to account for the potential influence of baseline depression severity, we repeated the analysis with baseline HAMD score as a regressor and obtained equivalent results.

Correlational analysis between described above connections (parameter estimates) and percent change in BDI score–total score and score on its subscales (affective, cognitive and somatic), as well as STAI-Trait total score, showed significant correlations between both right and left FPN and percent change in BDI score–total and on subscales, as well as total STAI-Trait score. No significant correlations were observed for the Somatomotor-Auditory Network and DMN, except for the borderline correlation between a change in BDI affective subscale score and DMN connectivity. Details of the correlations are provided in Table 3. There were no significant correlations between baseline HAMD, BDI (total score and subscales) and STAI-Trait scores, as well the length of the episode (see Supplementary Table 2 for details).

**Table 3:** Correlations between established functional connectivity measures (parameter estimates) and clinical scores (% change baseline vs. post-treatment). *HAMD*, Hamilton Depression Rating Scale; *BDI*, Beck Depression Inventory (BDI) (Beck et al. 1961); *STAI-T*, Spielberger’s State-Trait Anxiety Inventory–Trait

**Correlations**		**HAMD**	**BDI Total**	**BDI Affective**	**BDI Somatic**	**BDI Cognitive**	**STAI-T**
**Pearson's r**		**% Difference**	**% Difference**	**% Difference**	**% Difference**	**% Difference**	**% Difference**
	Correlation Coefficient	− 0.601	− 0.614	− 0.564	− 0.578	− 0.556	− 0.464
**Right FPN**	Sig. (2-tailed)	< 0.001	< 0.001	0.001	< 0.001	0.001	0.006
	*N*	34	34	34	34	34	34
	Correlation Coefficient	− 0.412	− 0.376	− 0.394	− 0.297	− 0.366	− 0.491
**Left FPN**	Sig. (2-tailed)	0.015	0.029	0.021	0.088	0.033	0.003
	*N*	34	34	34	34	34	34
**Sensorimotor**	Correlation Coefficient	− 0.365	− 0.321	− 0.294	− 0.316	− 0.303	− 0.239
**Auditory**	Sig. (2-tailed)	0.034	0.064	0.092	0.068	0.081	0.173
**Network**	*N*	34	34	34	34	34	34
	Correlation Coefficient	0.371	0.327	0.341	0.32	0.253	0.29
**DMN**	Sig. (2-tailed)	0.031	0.059	0.048	0.065	0.149	0.096
	*N*	34	34	34	34	34	34

Functional connectivity between:

-right fronto-parietal network (FPN) and left postcentral gyrus, left precentral gyrus, bilateral precuneus and left somatosensory association cortex

-left FPN and left postcentral/precentral gyri

-sensorimotor-auditory network and right supramarginal gyrus, right angular gyrus, right precuneus and posterior cingulate cortex

-posterior default mode network (DMN) and right angular and supramargial gyri

## Discussion

The main aim of the study was to explore baseline (i.e. pre-treatment) patterns of resting-state functional connectivity (rsFC) associated with response to SSRI treatment. To achieve this, we compared pre-treatment rsFC in depressed individuals who responded to 6 weeks of treatment with escitalopram, depressed individuals who did not respond to such treatment and rsFC in untreated healthy individuals without depression.

Using an unbiased ICA approach, we identified a number of patterns. Treatment responsivity was associated with enhanced rsFC of the right FPN—with the posterior DMN (bilateral precuneus), and with the SMN and somatosensory association cortex. At the same time, treatment non-responders appeared to be characterized by reduced rsFC: of the bilateral FPN with the contralateral SMN, of the right FPN with the posterior DMN (bilateral precuneus), and of the wide sensorimotor-auditory area (including the SMN, auditory cortex, posterior insula, central and parietal operculum, MCC, and SMA) with ANG/SMG and the posterior DMN (precuneous and PCC). We also observed reduced rsFC of the posterior DMN with ANG/SMG in treatment responders, although only when compared with HC.

This brief summary of results points at interactions between three networks—the DMN, the FPN, and primary sensory/motor regions—as being key to response to escitalopram in our study.

Our results suggest that the DMN, and in particular its posterior part, may be significant for treatment response, both when its connectivity as a network, and connectivity of its elements with other networks, is considered. Firstly, treatment R, as compared to NR, showed greater rsFC between the right FPN and the posterior DMN hub, precuneus, while there was no difference between R and HC (Fig. 2). Secondly, significantly reduced rsFC of the posterior DMN with the right ANG/SMG was observed in R compared to HC (Fig. 4), and was generally reduced in patients compared to HC (results not presented). Thirdly, significantly reduced rsFC of the extended somatomotor-auditory area including—mostly primary—sensory and motor regions, with the posterior DMN hub in the precuneus/PCC, and with the ANG/SMG, was seen in NR compared to HC (Fig. 3c), and generally in all patients versus HC (results not presented).

The DMN, in which abnormalities have been consistently observed in depression, is a network involved in a wide array of inward-directed mental activities, including self-referential processing, imagery and memory (Buckner et al. 2008). The involvement of the DMN is one of the most consistent findings regarding the physiopathology of depression (Dutta et al. 2014) and normalization of increased rsFC between key regions of the DMN may underlie antidepressant treatment response (McCabe et al. 2011). The DMN is organized into subsystems, which converge on the mPFC (anterior DMN) and precuneus/PCC (posterior DMN) (Buckner et al. 2008). While both parts of the DMN are involved in self-related processing, the anterior DMN shows greater bias towards self-other relations, and the posterior DMN—towards self-centred processing; it also has a role in involuntary (bottom-up) attentional processing, in particular related to visual and sensorimotor stimuli. The posterior DMN might have a particular role in treatment response, as suggested by our study and research by other groups, which observed normalization of the posterior DMN connectivity after successful antidepressants treatment (Li et al., 2013; Posner et al., 2013; Wang et al. 2014; Shen et al. 2015), while that of the anterior DMN remained unaffected (Li et al. 2013).

The FPN, composed of lateral prefrontal and posterior parietal cortices, is highly integrated with multiple brain networks, which allows a flexible and coordinated higher order modulation of cognitive and emotional processes (Marek and Dosenbach 2018, 2019). Our results show that greater temporal coherence between the FPN and the precuneus, similar to healthy individuals, was associated with favourable treatment response, which suggests that sustained FC between these structures may be essential for antidepressant response. This may be linked to control that the FPN exerts over the DMN (Cavanna and Trimble 2006; Chen et al. 2013; Marek and Dosenbach, 2019). FPN-DMN interactions are facilitated by rich cortico-cortical connections between the frontal and parietal lobes and, specifically, precuneus (Cavanna and Trimble 2006). Our findings are in line with already published research (e.g. Posner et al. 2013).

Good response to treatment was also associated with reduced connectivity of the DMN with the right ANG/SMG. The ANG/SMG are parts of the inferior parietal lobule (IPL), a complex region involved in complex functions across cognitive and emotional domains and supporting inter-network interactions thanks to hosting the nodes of major brain networks (Seghier 2013, Ingelström and Graziano, 2017). The area identified in our study corresponds to the location of the DMN node. The ANG and DMN activities are often found to be correlated, and our findings suggest that a decrease in this correlation may be meaningful for treatment response. The IPL shows strong lateralization of function, with the right IPL, identified in our study, particularly involved in attentional processing (Ingelström and Graziano, 2017). One hypothesis explaining better treatment response is that this reduced connectivity might facilitate directing attention away from the DMN and inward-directed processing, and towards other functions supported by the IPL. The role for the right ANG in treatment response has been reported in previous studies (Guo et al. 2013; Korgaonkar et al. 2014), with one showing its best performance in classifying the early improvement status among other identified, DMN and frontal, regions.

The third finding involving DMN in our study was that treatment NR were characterized by significantly decreased FC of the sensorimotor-auditory component extending across the SMN, primary auditory cortex (Heschl’s gyrus and planum temporale), posterior insula, central and parietal operculum, MCC and SMA. Reduced temporal correlation was observed with the posterior DMN (the precuneus and PCC), and the right ANG/SMG in the cluster partly overlapping with the one described in the preceding paragraph. The component extends across canonical resting-state networks and it might be argued that it would further subdivide if dimensionality was increased. However, the common functional denominator of the areas involved is their role in sensory and motor processing, which supports treating it as a functional entity. The areas constituting this component include, mostly primary, somatosensory and somatomotor areas, which, through perception and integration of the internal and external sensory data (related to touch, temperature, pain, proprioception, interoception, visceral sensations, as well as auditory, olfactory and gustatory stimuli) facilitate planning of complex and coordinated motor movements, their production, execution and control (Northoff 2016). These areas are also strongly involved in production of emotional states (Kropf et al. 2019). It has been proposed that sensory information from both internal and external environments is one of the key elements in emotion formation and that internal body sensations influence the subjective experience of emotion (Levenson 1999; Wiens 2005; Pace-Schott et al. 2019). Our results suggest that a certain level of sustained connectivity of these sensory and motor regions, with the posterior DMN and the ANG/SMG cluster in the IPL, is important for treatment response. This may be related to the role of posterior DMN and the right IPL in involuntary attentional processing, crucial for appropriate processing of the sensorimotor stimuli (Seghier 2013). Additionally, this component was also identified in another recent study using a data driven-approach (Martens et al. 2021). Additionally, this component was also identified in another recent study using a data driven-approach (Martens et al. 2021).

Importance of the sensory and motor processing, as well as of the FPN, for treatment response was again supported by an association of the failure to respond to escitalopram with reduced rsFC of the bilateral FPN with contralateral precentral and postcentral gyri, as compared to both R and HC, and with somatosensory association cortex, as compared to R. The FPN provides flexible modulation of the activity of other brain regions and its inadequate connectivity with primary and associative somatosensory and motor regions may affect how well this control will be exerted (Marek and Dosenbach, 2018, 2019). In depression, reduction in the SMN resting-state neuronal variability was linked to decreased modulation of spontaneous activity in the SMN by external stimuli (Martino et al., 2016; Conio et al. 2019). Apart from the importance of somatosensory processing for emotion formation (Kropf et al. 2019), at the behavioural level, this might translate into reduced responsiveness and psychomotor activation, common symptoms of depression (Martino et al. 2016). Aberrant FC of the CEN (corresponding to the FPN) with the pre- and postcentral gyri in depression was observed using the ICA approach (Dichter et al. 2015), and the role of FPN-SMN connectivity for treatment response was recently shown in a large multicentre study (EMBARC) (Chin-Fatt et al. 2020). Our study adds to the growing body of results suggesting a role for the somatosensory and motor processing in antidepressant response.

Previous research on the role of resting-state connectivity for treatment response in depression yielded a range of results, and although they are largely heterogeneous, a few general themes were suggested (for detailed review of findings see Dichter et al. 2015; Brakowski et al. 2017). The most consistent pattern was an association of treatment response with an increase in connectivity between frontal and limbic areas, in line with the importance of cognitive control over emotional circuits. We did not observe this in our study. Other themes included the role for visual recognition circuits and subgenual cingulate cortex connectivity. The review also suggested that treatment-sensitive patients may have lower connectivity within the DMN (Guo et al., 2012; Ma et al., 2012). More recent studies strongly provided further support for the role of the DMN and FPN in treatment response; interestingly, an involvement of the SMN and sensory regions has been a more common finding, reported, for example, by the large multicentre studies iSPOT (Korgaonkar et al. 2019) and EMBARC (Chin-Fatt et al. 2020). ISPOT indeed identified functional connectivity between the DMN, the FPN and the SMN as particularly important for treatment responsiveness, suggesting that elevated connectivity was generally associated with better treatment response, while hypoconnectivity at baseline distinguished NR from HC. This was in line with our findings, as failure to respond was generally associated with reduced connectivity as compared to HC. Despite emerging themes, findings vary largely between the studies. This heterogeneity may be caused by group differences regarding, for example, symptomatic pictures, chronicity, treatment status (e.g. drug-naive, drug-free, under treatment), or the length and type of treatment. Not less important are technical aspects, such as differences in data collection methods (e.g. the length of resting-state data acquisition, eyes-open vs. eyes-closed paradigms), and differences in data analysis approaches (e.g. ICA or seed-based, ReHo, graph theory). The picture becomes even more complex when not only network involvement is considered, but also details of the affected connectivity, e.g. related to the structures, with which network connectivity are altered. To address these questions, a larger number of studies, providing all their findings, followed by meta-analyses, are needed.

In this study, we performed post hoc correlational analysis to explore potential relationships between the extracted functional connectivity measures and subjective measures of mood and anxiety. The primary aim of both BDI and STAI is to measure the global construct of, respectively, depression and anxiety. Previous research however showed that BDI contains items that can be grouped as separate factors, which not only may be more sensitive to change (Beck et al., 1996) but, in line with dimensional approach, may also have different neural correlates, which may have bearing on treatment response (Nicholson and Sommer, 2018). Given that HAMD score change was used to define response, and FC measures assessed already survived multiple comparisons at the whole-brain level, significant correlations were expected for the change in HAMD score—and strongly correlated with it total BDI score. At the same time, correlations between change in scores in BDI subscales (affective, cognitive and somatic), and STAI-Trait total score, have the potential to provide new information about the relationship between distinctive groups of symptoms, their improvement and particular connections. Our data points at the particular role of both the right and left FPN connectivity—likely reflecting better cognitive control—for response across BDI dimensions and STAI. Interestingly, we were unable to show any relationship between the sensorimotor-auditory network and anxiety or somatic dimension of BDI. The results suggest that considering separate factors of multidimensional scales may have value for a better understanding of the role of particular connections in the context of specific symptomatic dimensions. It needs to be noted, however, that this is a post hoc exploratory analysis on a small group, and the subject would need to be explored in studies specifically designed to test it, using scales validate for testing some groups of symptoms, such as State-Trait Inventory for Cognitive and Somatic Anxiety (STICSA) (Grös et al. 2007).

Our study has both strengths and limitations. The main limitation is the small size of the group, which reduces the power and increases the likelihood of type 2 error. Also, prediction analysis, e.g. cross-validation, was not performed due to the limited sample size, which restricts the interpretation of the data. In the future, a replication of findings in a larger independent sample will be needed. An important strength was that the group consisted of carefully selected drug-free individuals with depression, who underwent an assessment by the same psychiatrist to increase homogeneity. Treatment was standardized, with the same medication and dose for all participants. This is a strength of the study, although it also means that conclusions can be drawn for escitalopram only. Another strength was related to analytical methods chosen. Our study is one of the few using ICA rather than seed-based approach. This allowed for an unbiased exploration of functional networks, not restricted by existing hypotheses. A limitation of such an approach is however the lack of exploration of rsFC of the structures, which role has been shown by other studies, yet which may not pass the statistical threshold, which is more stringent in whole-brain analysis than in a seed-based approach. The stringent statistical approach using nonparametric permutation testing and TFCE (Threshold-Free Cluster Enhancement) allows further confident interpretation of the results. However, it needs to be noted that due to the exploratory nature of this study, correction for multiple comparisons related to inclusion of multiple RSNs was not performed. One general limitation, shared by all functional connectivity studies, is related to the very nature of this approach, where the estimation of the functional connectivity is based on the temporal correlation of activities in various brain regions and does not provide information about anatomical connections nor directionality of the effect or causality. Hypotheses regarding the meaning of findings may however be proposed based on known anatomy and function, as well as interactions of structures involved.

In summary, patterns of resting-state functional connectivity related to escitalopram treatment response focused primarily on three networks, the DMN, the FPN and the SMN. This adds to the body of results supporting importance of network connectivity for treatment response. Future studies are needed that will meta-analyze data from different studies, accounting for potential sources of heterogeneity of the results.

## Supplementary Information

Below is the link to the electronic supplementary material.

Supplementary file1 (DOCX 18 KB)

Supplementary file2 (DOCX 22 KB)
