# Supplementary material for: Resting state functional connectivity patterns as biomarkers of treatment response to escitalopram in patients with major depressive disorder
Source: Psychopharmacology (Berl). 2021 Sep 3;239(11):3447–60. doi: 10.1007/s00213-021-05915-7 (PMC9584978; doi:10.1007/s00213-021-05915-7)
Supplement: Supplementary file 1 — Supplementary file1 (DOCX 18 KB) [file 213_2021_5915_MOESM1_ESM.docx]

Supplementary Table 1. List of items included in Beck Depression Inventory (BDI) subscales.

| Beck Depression Inventory | |
| --- | --- |
| Subscale | Items |
|  |  |
| Affective | 1 sadness  2 pessimism/being discouraged about future  4 loss of pleasure  9 suicidality  10 crying  12 loss of interest |
| Cognitive | 3 past failure  5 guilt feelings  6 punishment feeling  7 self-disappointment  8 self-criticism |
| Somatic | 11 irritability  15 loss of energy  16 changes in sleep  17 tiredness  18 decrease in appetite  19 weight loss  20 worries about health  21interest in sex |
