# Supplementary material for: Resting state functional connectivity patterns as biomarkers of treatment response to escitalopram in patients with major depressive disorder
Source: Psychopharmacology (Berl). 2021 Sep 3;239(11):3447–60. doi: 10.1007/s00213-021-05915-7 (PMC9584978; doi:10.1007/s00213-021-05915-7)
Supplement: Supplementary file 2 — Supplementary file2 (DOCX 22 KB) [file 213_2021_5915_MOESM2_ESM.docx]

Supplementary Table 2. Correlations between established functional connectivity measures (parameter estimates) and baseline clinical scores. HAMD - Hamilton Depression Rating Scale; BDI - Depression Inventory (BDI); STAI-Trait - Spielberger’s State-Trait Anxiety Inventory - Trait

Functional connectivity between:

- Right FPN and left postcentral gyrus, left precentral gyrus, bilateral precuneus and left somatosensory association cortex
- Left FPN and left post + precentral gyri
- SAN and right supramarginal gyrus, right angular gyrus, right precuneus and posterior cingulate cortex
- Posterior DMN and right angular and supramargial gyri

| **Correlations** |  | **HAMD** | **BDI Total** | **BDI Affective** | **BDI Somatic** | **BDI Cognitive** | **STAI-T Total** | **Length episode** |
| --- | --- | --- | --- | --- | --- | --- | --- | --- |
| **Pearson's r** |  | **Score visit 1** | **Score visit 1** | **Score visit 1** | **Score visit 1** | **Score visit 1** | **Score visit 1** | **months** |
|  | Correlation Coefficient | 0.02 | -0.045 | -0.077 | 0.129 | -0.156 | -0.11 | -0.067 |
| **Right FPN** | Sig. (2-tailed) | 0.912 | 0.802 | 0.665 | 0.467 | 0.379 | 0.537 | 0.706 |
|  | N | 34 | 34 | 34 | 34 | 34 | 34 | 34 |
|  | Correlation Coefficient | -0.166 | -0.28 | -0.259 | -0.132 | -0.242 | -0.333 | -0.264 |
| **Left FPN** | Sig. (2-tailed) | 0.348 | 0.109 | 0.14 | 0.456 | 0.168 | 0.054 | 0.131 |
|  | N | 34 | 34 | 34 | 34 | 34 | 34 | 34 |
| **Sensorimotor** | Correlation Coefficient | 0 | 0.002 | -0.014 | -0.077 | 0.097 | -0.067 | 0.032 |
| **Auditory** | Sig. (2-tailed) | 0.999 | 0.991 | 0.936 | 0.663 | 0.586 | 0.708 | 0.856 |
| **Network** | N | 34 | 34 | 34 | 34 | 34 | 34 | 34 |
|  | Correlation Coefficient | 0.041 | -0.199 | -0.058 | -0.179 | -0.208 | -0.051 | 0.039 |
| **DMN** | Sig. (2-tailed) | 0.818 | 0.259 | 0.743 | 0.312 | 0.237 | 0.776 | 0.827 |
|  | N | 34 | 34 | 34 | 34 | 34 | 34 | 34 |
